# Luteolin as a therapeutic option for multiple sclerosis

**DOI:** 10.1186/1742-2094-6-29

**Published:** 2009-10-13

**Authors:** Theoharis C Theoharides

**Affiliations:** 1 Molecular Immunopharmacology and Drug Discovery Laboratory, Department of Pharmacology and Experimental Therapeutics, Tufts University School of Medicine and Tufts Medical Center, Boston, MA, USA; 2 Department of Internal Medicine, Biochemistry and Psychiatry, Tufts University School of Medicine and Tufts Medical Center, Boston, MA, USA

## Abstract

Multiple sclerosis (MS) remains without an effective treatment in spite of intense research efforts. Interferon-beta (IFN-β) reduces duration and severity of symptoms in many relapsing-remitting MS patients, but its mechanism of action is still not well understood. Moreover, IFN-β and other available treatments must be given parenterally and have a variety of adverse effects. Certain naturally occurring flavonoids, such as luteolin, have anti-oxidant and anti-inflammatory effects, including inhibition of activated peripheral blood leukocytes from MS patients. Luteolin also inhibits mast cells, as well as mast cell-dependent T cell activation, recently implicated in MS pathogenesis. Moreover, luteolin and structurally similar flavonoids can inhibit experimental allergic allergic encephalomyelitis (EAE), an animal model of MS in rodents. An appropriate luteolin formulation that permits sufficient absorption and reduces its metabolism could be a useful adjuvant to IFN-β for MS therapy.

## Introduction

This issue includes an interesting article by Sternberg et al. showing that the flavonoid luteolin inhibits IL-1, TNF and metalloproteinase-9 (MMP-9) release from activated peripheral blood mononuclear cells (PBMCs) from multiple sclerosis (MS) patients, and that the effect of luteolin is augmented by concurrent administration of interferon-beta (IFN-β). This paper extends previous similar results with quercetin that required higher concentrations of the flavonoid [1].

## Discussion

**Figure 1:**
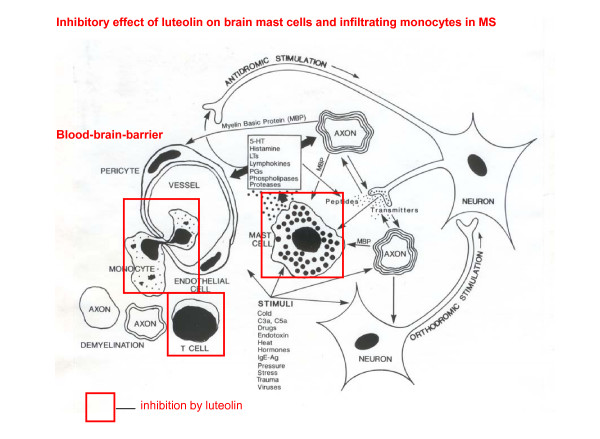
**Diagrammatic representation of the inhibitory effect of luteolin on brain mast cells and infiltrating monocytes in the pathogenesis of multiple sclerosis**.

Luteolin with or without IFN-β, could be helpful in MS by not only inhibiting PBMC release of cytokines, but also by inhibiting T cells, which we recently showed can be superstimulated by mast cells, an action also inhibited by luteolin [2]. In addition to T cells, recent evidence implicates also TH2 processes typically associated with allergic reactions [3-5], which involve mast cells (Fig. 1). In fact, mast cells have been considered as the next target for MS therapy [6-8].

Brain MS plaques also contain activated mast cells [9,10], which have been associated with brain demyelination [11-13]. Gene array analysis also showed that MS plaques had increased gene expression for the IgE receptor (FcεRI), the histamine-1 receptor and the protease tryptase, all of which are associated with mast cells [14-16]. Mast cell tryptase is elevated in the CSF of MS patients [17], can activate peripheral mononuclear cells to secrete TNF and IL-6 [18], as well as stimulate protease-activated receptors (PAR) to induce widespread inflammation [19]. Brain mast cells can secrete TNF [20], which is involved in both brain inflammation [21] and blood-brain-barrier (BBB) permeability [22]. In fact, BBB disruption precedes any pathologic signs of MS [23] and mast cells can disrupt the BBB [24,25].

Flavonoids such as quercetin have potent anti-oxidant and anti-inflammatory activity [26]. Quercetin and luteolin also inhibit human cultured mast cell release of histamine, leukotrienes and prostaglandin D_2 _[27], as well as IL-6, IL-8, TNF-α and tryptase [28,29]. Moreover, quercetin and luteolin inhibit mast cell activation stimulated by IL-1 [30] leading to selective release of IL-6. Luteolin also inhibits IL-6 release from microglia cells [31], and from astrocytes [32]. Flavonoids can also inhibit myelin phagocytosis by macrophages [33], as well as inhibit EAE [34-36].

## Conclusion

Quercetin and its structurally related luteolin are safe [37]. The fact remains that less than 10% of flavonoids are absorbed orally [37]. Novel ways of delivering select flavonoid combinations would be required to assure sufficient plasma levels, especially if luteolin were to also inhibit brain inflammation. Such a test nutraceutical formulation has already been tried on a number of relapsing-remitting MS patients treated with INF-β with encouraging positive results.

## Competing interests

TCT has been awarded US patents No 6,689,748; 6,984,667 and EPO No 1365777 that cover the use of flavonoids in inflammatory diseases; he has also filed (3/30/04) US patent applications No. 10/811,826; 11/214,831; 11/999,991; 12/151,268 specifically covering combinations of flavonoids, including luteolin with INF-β, for the treatment of MS.
